# Editorial to “disparities in cardiac arrest mortality among patients with chronic kidney disease: A US based epidemiological analysis”

**DOI:** 10.1002/joa3.70008

**Published:** 2025-02-03

**Authors:** Masamichi Yano

**Affiliations:** ^1^ Division of Cardiology Osaka Rosai Hospital Sakai Osaka Japan

We appreciated the report by Shahid et al., which underscores chronic kidney disease (CKD) as a significant risk factor for cardiovascular disease (CVD), with cardiac arrest (CA) emerging as a leading cause of death among patients with impaired renal function.^1^ This study provides a detailed analysis of CKD‐related CA mortality trends in the United States over two decades, using data from the Centers for Disease Control and Prevention (CDC). Notably, it highlights persistent disparities in mortality across sex, racial/ethnic, and geographic subpopulations, with the Social Vulnerability Index (SVI) emerging as a critical determinant of excess mortality. These findings stress the urgent need for interventions targeting healthcare inequities in CKD and cardiovascular outcomes.

CKD has long been recognized as a major risk factor for CVD, with CA being a predominant cause of mortality in renal dysfunction patients.^2^ The underlying pathophysiology involves electrolyte imbalances, autonomic dysfunction, systemic inflammation, and structural changes in the heart. CKD and CVD mutually exacerbate each other, increasing arrhythmic susceptibility, particularly in patients with advanced renal impairment.^3^ Despite advancements in renal and cardiovascular care, the persistently high mortality rates among CKD patients experiencing CA underscore the need for more effective preventive and therapeutic strategies.

The study reveals that the age‐adjusted mortality rate (AAMR) for CKD‐related CA has remained stable over the past two decades, while general mortality has declined. This suggests potential gaps in risk factor management and the implementation of evidence‐based interventions for CKD patients. It also highlights significant disparities across demographic and geographic groups. Men had higher mortality rates than women, possibly due to differences in CKD progression, cardiovascular risk, and healthcare access. Racial and ethnic disparities were evident, with non‐Hispanic (NH) Black and Hispanic populations experiencing disproportionately high CA‐related mortality. Socioeconomic factors, captured by the SVI, were critical in these disparities, highlighting the role of social determinants of health (SDoH) like income inequality, healthcare access, education, and neighborhood safety. While NH Black populations had the highest mortality rates, the study reports a decline in CA‐related mortality among them, likely due to improvements in healthcare access and CKD management. However, more efforts are needed to extend these gains to other high‐risk groups. The study also identifies regional disparities, with urban areas showing higher mortality rates than rural regions. Although urban areas have better access to healthcare, environmental stressors like air pollution and socioeconomic deprivation may exacerbate cardiovascular risk. Surprisingly, the highest mortality rates were seen in the Western U.S., challenging the conventional view that the Southern region bears the greatest CVD burden. This warrants further investigation into regional factors.

A key contribution of the study is the inclusion of the SVI as a determinant of mortality disparities. The data reveal a stark gradient in CA‐related mortality across SVI quartiles, with the most vulnerable communities (Q4) facing significantly higher mortality rates than the least vulnerable (Q1). This emphasizes the impact of social determinants of health (SDoH) on outcomes for CKD patients. Socioeconomically disadvantaged communities encounter barriers to healthcare, including limited access, suboptimal disease management, and exposure to chronic stressors that elevate cardiovascular risk. Disparities in healthcare infrastructure, especially in low‐resource settings, worsen these challenges. The COVID‐19 pandemic amplified these disparities, disproportionately affecting females, Hispanic, NH Asian, and NH Black populations, as well as urban residents. This aligns with broader findings that COVID‐19 disproportionately impacted socially vulnerable groups, exacerbated by healthcare disruptions, economic instability, and psychosocial stress. The study's findings suggest a multifaceted approach to addressing CKD‐related CA mortality disparities. Strategies should include enhanced screening and early detection programs for high‐risk populations. Integrating renal and cardiovascular care in primary healthcare settings could facilitate early intervention and improve outcomes. Expanding access to guideline‐directed medical therapy for CKD and arrhythmia management is essential for reducing CA risk. Addressing SDoH through policy‐driven interventions, such as expanding health insurance, improving preventive healthcare access, and investing in community health initiatives, is vital for reducing inequities in CKD management. Addressing environmental and lifestyle factors contributing to urban mortality disparities should focus on urban planning, air quality regulations, and public health campaigns. Further research is needed to explore the genetic underpinnings of racial disparities in CKD‐related CA, such as the role of APOL1 variants in Black population. This could lead to personalized medicine approaches combining genetic risk stratification with SDoH metrics, allowing clinicians to develop tailored treatment strategies considering both biological and socioeconomic determinants. Longitudinal studies are needed to examine the impact of healthcare interventions, policy changes, and community programs on CKD outcomes. Incorporating machine learning and big data analytics into epidemiological research could improve predictive modeling and risk stratification. Public health initiatives should prioritize education and awareness campaigns to promote early CKD detection and adherence to cardiovascular preventive measures. Collaboration between healthcare providers, policymakers, and community organizations will be essential to implement sustainable solutions to reduce CKD‐related CA mortality.

In conclusion, Shahid et al.'s study underscores the urgent need to address disparities in CKD‐related CA mortality, with SVI emerging as a key determinant of excess mortality. Persistent racial, ethnic, and geographic inequities highlight the need for targeted interventions that integrate medical, social, and policy‐driven approaches. By leveraging a comprehensive understanding of CKD and CA epidemiology, healthcare systems can implement strategies to promote equitable health outcomes and improve survival among vulnerable populations. Future research should continue exploring innovative solutions to bridge gaps in CKD care and mitigate cardiovascular disparities, ultimately fostering a more inclusive healthcare system.

## FUNDING INFORMATION

The author has nothing to report.

## CONFLICT OF INTEREST STATEMENT

The authors declare no conflicts of interest.
